# Dietary Differentiation Among Sympatric Sri Lankan Bats Revealed by Nanopore Metabarcoding

**DOI:** 10.1002/ece3.74545

**Published:** 2026-11-13

**Authors:** Thejanee Perera, Sahan Siriwardana, Inoka Perera, Shiroma Handunnetti, Jagathpriya Weerasena, Gayani Premawansa, Sunil Premawansa, Wipula Yapa, Andreas Nitsche, Claudia Kohl

**Affiliations:** ^1^ Institute of Biochemistry, Molecular Biology and Biotechnology University of Colombo Colombo Sri Lanka; ^2^ Highly Pathogenic Viruses (ZBS1), Centre for Biological Threats and Special Pathogens Robert Koch Institute Berlin Germany; ^3^ Department of Zoology and Environment Sciences, Faculty of Science University of Colombo Colombo Sri Lanka; ^4^ Colombo North Teaching Hospital Ragama Sri Lanka

**Keywords:** bats, dietary analysis, disease vectors, metabarcoding, nanopore sequencing, pest control, Sri Lanka

## Abstract

Understanding trophic partitioning within multispecies bat assemblages is essential for evaluating their ecological roles in agroecosystems and disease‐vector regulation. We assessed the diets of four sympatric cave‐roosting bats in Sri Lanka—*Hipposideros speoris, Miniopterus phillipsi, Rhinolophus rouxii*, and *Rousettus leschenaultii* —using mitochondrial cytochrome b metabarcoding on the Oxford Nanopore platform. Across 22 fecal samples, we detected a diverse assemblage of *Lepidoptera, Diptera, Coleoptera*, and *Hemiptera*, including multiple agriculturally significant pest genera (e.g., *Spodoptera, Cnaphalocrocis*) and medically relevant vectors (*Aedes, Anopheles, Phlebotomus*). Preliminary data suggests *Lepidoptera* dominated the diets of *M. phillipsi* and *R. rouxii* , whereas *H. speoris* consumed proportionally more dipterans. Notably, insect DNA was detected in the frugivorous *R. leschenaultii* , may suggest facultative insectivory or incidental ingestion. Although statistical power was limited by uneven sample sizes and amount of samples, the results are suggestive of potential trophic differentiation among sympatric taxa. These findings provide baseline molecular dietary data for Sri Lankan bats and support their potential contribution to agroecosystem functioning and vector regulation within a One Health framework. However, this pilot study uses a snapshot sampling approach and can provide first insights and the baseline for further studies.

## Graphical abstract

Dietary composition and prey–bat associations in Sri Lanka.

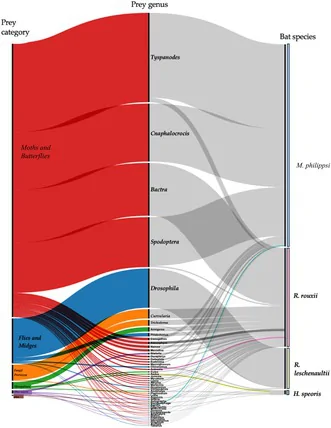

## 1 Introduction

Bats are a distinctive group of mammals occupying a remarkable diversity of ecological niches, making them one of the most taxonomically and functionally diverse groups of mammals worldwide (Kunz 1982). With over 1200 species described across the globe, bats have evolved unique morphological, physiological, and behavioral adaptations that allow them to exploit a wide array of habitats and food resources (Kunz 1982). In Sri Lanka alone, more than 30 bat species are reported, inhabiting forests, caves, agricultural landscapes, and urban areas (Yapa and Ratnasooriya 2006). These species exhibit distinct dietary preferences and foraging strategies, providing an ideal framework for ecological and molecular studies on predator–prey interactions, ecosystem functioning, and biodiversity assessment (Perera, Schwarz, et al. 2022).

As key components of ecosystems, bats contribute to a variety of essential ecological services, including insect population control, seed dispersal, and pollination (Ramírez‐Fráncel et al. 2022). Their feeding habits are remarkably diverse, spanning insectivory, frugivory, nectarivory, carnivory, piscivory, and sanguivory (Clare and Oelbaum 2024). Insectivorous bats, in particular, consume substantial quantities of nocturnal insects—sometimes exceeding 30% of their body weight in a single night—thereby reducing populations of agricultural pests, lowering crop losses, and limiting the spread of vector‐borne diseases (Kunz 1974). Studies have demonstrated that bat predation on crop pests, such as moths and beetles, can significantly enhance agricultural productivity and contribute to natural pest suppression, offering a sustainable alternative to chemical pesticides (Tuneu‐Corral et al. 2023). Meanwhile, frugivorous and nectar‐feeding bats function as keystone pollinators and seed dispersers, enhancing plant reproduction, maintaining genetic diversity, and promoting forest regeneration. Certain plant species, including economically and ecologically important taxa such as durian ( *Durio zibethinus* ) and baobab (*Adansonia* spp.), are entirely dependent on bats for pollination and seed dispersal (Muscarella and Fleming 2007; Aziz et al. 2021). These roles position bats as keystone species, critical for maintaining ecosystem resilience, biodiversity, and ecological stability.

Bats' evolutionary adaptations for powered flight, including specialized wing morphology, high metabolic rates, and short, rapid‐transit digestive tracts, confer efficient energy extraction from nutrient‐rich diets but also create a dependency on high‐quality food sources (Speakman et al. 2003). Diet not only supports these energetically demanding lifestyles but also shapes gut microbiome composition and function, influencing nutrient assimilation, immune function, and overall health, which in turn affects bats' roles in ecosystems (Bazzoni et al. 2024). Understanding dietary ecology is therefore essential for evaluating the broader ecological contributions and conservation needs of these species.

Over the past decade, molecular methods have transformed dietary studies by offering unparalleled taxonomic resolution and sensitivity compared to conventional observational approaches (Ingala et al. 2021). Next‐generation sequencing (NGS) technologies allow researchers to detect and identify prey species at fine taxonomic scales, including cryptic or nocturnal taxa that are difficult to observe directly (Garg et al. 2023). Among the most widely used molecular markers for dietary analyses is the mitochondrial cytochrome b (Cytb) gene, which facilitates species‐level identification of prey and provides insights into trophic interactions (Pickett et al. 2012). The combination of Oxford Nanopore sequencing technology with Cytb amplicon analysis offers a rapid, cost‐efficient, and scalable approach to profile bat diets, providing both depth and breadth of dietary information across multiple sympatric species.

In this study, we focus on four bat species— *Hipposideros speoris* , *Miniopterus phillipsi*, *Rhinolophus rouxii* , and *Rousettus leschenaultii* —cohabiting a natural cave in Sri Lanka. These species were selected to examine dietary composition and lay the basis for exploring patterns of niche partitioning, prey overlap, and functional complementarity in the future. By profiling prey diversity across sympatric bats, this study aims to provide first insights into the ecological services these species provide, including agricultural pest suppression, vector regulation, and contributions to ecosystem stability.

Importantly, molecular dietary analyses not only enhance our understanding of bat–ecosystem interactions but also underscore the conservation value of bats (Gonçalves et al. 2023). Quantifying their diets allows for a direct appreciation of the ecosystem services they provide, which can inform targeted conservation strategies, habitat management, and policy decisions. Given the threats bats face from habitat loss, roost disturbance, and anthropogenic pressures, detailed knowledge of their feeding ecology is crucial for safeguarding both bat populations and the ecological functions they support.

In summary, this study uses advanced molecular tools to characterize the diets of multiple sympatric bat species in a Sri Lankan cave system, bridging the gap between ecological research and conservation application. By linking dietary patterns to ecosystem services, this work provides first insights into the role of bats in maintaining agricultural productivity, mitigating vector‐borne disease risks, and supporting biodiversity, emphasizing the need to integrate dietary ecology into bat conservation frameworks.

## 2 Methodology

### 2.1 Sampling Period and Study Location

Bat sampling was conducted from 21 to 25 January 2019 at Wavulgalge cave, located in Koslanda, Badulla district, Sri Lanka (6°43′37.1676″N, 81°3′11.7216″E).

Bats were captured using hand nets during their emergence from the cave. Each individual was examined and recorded for age, gender, and forearm length. Age was categorized as juvenile, sub‐adult, or adult, while gender was determined based on external sexual characteristics as described before (Perera, Siriwardana, et al. 2022).

### 2.2 Ethical Considerations and Safety Measures

The study was conducted in compliance with ethical standards, with approval from the Department of Wildlife Conservation, Sri Lanka (Permit No. WL/3/2/05/18) and the Institute of Biology, Sri Lanka (ERC IOBSL 170 01 18). Bats were captured using hand nets, handled with gloved hands, and sampled quickly to minimize stress before being released at the capture site. Laboratory analyses were performed under BSL‐2 conditions at the Robert Koch Institute, Berlin, Germany, in accordance with strict biosafety protocols to reduce anthroponotic and zoonotic risks.

### 2.3 Biological Sample Collection

Oral swab samples were collected with sterile swabs from four sympatric Sri Lankan cave bat species (*Hipposideros speoris, Miniopterus phillips, Rhinolophus rouxii*, and *Rousettus leschenaultii* ) for accurate species identification using cytochrome b gene analysis (Figure S1 and Table 1). For dietary analysis, fecal pellets were collected directly during animal handling. Samples were transferred into sterile 2 mL microtubes and preserved in a VOYAGEUR (Air Liquide, Austria) dry shipper containing absorbed liquid nitrogen to maintain the cold chain during transport. Further details on sample collection and processing are available in the publication “One Health Approach for the Sampling of Different Bat Species Living in a Sympatric Colony” (Perera, Siriwardana, et al. 2022).

**TABLE 1:** Primer sequences of the PCR assay. Binding sites are visualized in Figure S1.

Primer name	Primer sequence (5′–3′)	Amplicon length (bp)
Forward CB 3	GAGGAGCAACTGTAATTACTAA	358
Reverse CB 4	AAAAGAAARTATCATTCAGGTTGAAT	

A total of 22 fecal samples were collected: 1 from *H. speoris* , 9 from *M. phillips*, 2 from *R. leschenaultii* , and 10 from *R. rouxii* (Table 2).

**TABLE 2:** Field sampling details of bat fecal sample pools used in dietary analysis.

Bat species	Individuals	Bat identification number	Capture date	Age class	Gender
*H. speoris*	2	380	1/25/2019	Adult	♂
*M. phillipsi*	5	332, 344, 346, 349, 351	1/23/2019	Adult	♂
	4	345, 347, 348, 350	1/23/2019	Adult	♀
*R. rouxii*	4	333, 335, 336, 337	1/23/2019	Adult	♀
	2	338, 339	1/23/2019	Adult	♂
	2	381, 384	1/25/2019	Adult	♂
	2	382, 383	1/25/2019	Adult	♀
*R. leschenaultii*	1	358	1/23/2019	Adult	♀
	1	372	1/24/2019	Sub‐Adult	♀

*Note:* Gender codes‐ ♀ for Female, ♂ for Male.

### 2.4 DNA Extraction and Bat Species Identification

DNA was extracted from fecal samples using the QIAamp Fast DNA Stool Mini Kit (Qiagen, Hilden, Germany), following the manufacturer's instructions. DNA yield was quantified using Qubit fluorometry, and samples were normalized to an input concentration of 5–10 ng/μL. Negative extraction controls were included to monitor for contamination.

For bat species identification, the full cytochrome b gene was amplified from oral swabs using RrFP and RrRP primers as previously described (Perera, Schwarz, et al. 2022). Individual bat species were confirmed using standard molecular identification protocols (Perera, Schwarz, et al. 2022).

### 2.5 Nanopore Sequencing—Library Preparation, Basecalling, and Demultiplexing

Purified PCR amplicons were used to prepare the sequencing library with the Ligation Sequencing Kit (SQK‐LSK109) and barcoded using the Native Barcoding Expansion 1–12 (EXP‐NBD104). DNA end‐repair and dA‐tailing were performed using the NEBNext End Repair/dA‐Tailing Module, followed by ligation of Oxford Nanopore sequencing adapters per manufacturer's instructions. Barcoded amplicons were pooled and loaded onto a SpotON FLO‐MIN106 flow cell and sequenced on a MinION Mk1C device (Oxford Nanopore Technologies, Oxford, United Kingdom). Real‐time basecalling and demultiplexing were performed using the FastQ basecalling model in MinKNOW software (v20.05.12).

### 2.6 Bioinformatic Analysis—Quality Control and Taxonomic Assignment

Raw Nanopore reads were assessed with FastQC (v0.12.1), and low‐quality bases were filtered and trimmed to 300–400 bp using fastp (v1.0.1). Host‐derived sequences were removed by aligning reads to the respective bat reference genomes using minimap2 (v2.28). Residual adapters and chimeric reads were removed with Porechop (v0.2.4). The resulting demultiplexed, host‐depleted reads were compared against the NCBI non‐redundant (NR) database using DIAMOND blastx (v2.1.13) (Huson et al. 2007; Buchfink et al. 2015). Taxa represented by ≥ 10 reads and passing an E‐value cutoff of 1e‐5 were retained for downstream analysis, ensuring that low‐abundance spurious matches were excluded while capturing true prey diversity.

### 2.7 Methodological Considerations

This study represents an exploratory pilot assessment with uneven and limited sample sizes. In particular, the single fecal sample available for *H. speoris* restricts inference regarding species‐level dietary breadth. Relative read abundance metrics derived from metabarcoding should not be interpreted as quantitative biomass proxies due to amplification bias, mitochondrial copy number variation, and differential prey digestion. Additionally, sampling occurred during a single dry‐season interval, precluding assessment of seasonal dietary shifts. Statistical power to detect moderate dietary overlap was limited, and non‐significant correlations should not be interpreted as definitive evidence of niche segregation. These constraints highlight the need for expanded temporal and sample replication in future investigations.

Potential sources of bias inherent in metabarcoding were considered, including PCR amplification bias due to primer mismatch, differential digestibility of prey, variation in mtDNA copy number among prey taxa, and retention‐time biases in the digestive tract. Negative PCR and extraction controls were included to monitor contamination. The minimum read threshold, input DNA concentration, and *E*‐value cutoff were selected to balance sensitivity and specificity, in line with established metabarcoding best practices.

## 3 Results

The bats inhabiting Wavulgalge Cave, Sri Lanka, and included in this pilot study were molecularly identified as *H. speoris* , *M. phillipsi* , *R. leschenaultii* , and *R. rouxii* ; the detailed identification has been published separately (Perera, Schwarz, et al. 2022) These small sample sizes reflect the pilot nature of this study and the snapshot sampling approach. Dietary composition for these species may not represent population‐level patterns, and results should be interpreted accordingly.

### 3.1 Sequencing Output

The nanopore sequencing run resulted in an average of 176,683 raw reads per sample pool. Following quality control and filtering with fastp, an average of 175,535 reads was retained, corresponding to a 99.5% retention rate and reflecting the high quality of the raw sequencing data. Number of reads aligning to a given taxon; only samples exceeding 1000 reads were included in the analysis, including those assembled into contigs that mapped to the taxon.

### 3.2 Allocation of Sequencing Reads to Prey Species

Dietary profiling could indicate distinct prey preferences among the four bat species (Table 3 and Supplementary Table S1). Given the limited sample numbers the results are preliminary: *R. rouxii* exhibited the widest prey spectrum, dominated by mosquitoes and moths, while *H. speoris* also consumed substantial numbers of mosquitoes together with cockroaches and ants. *M. phillips may* showed a strong bias toward moth‐rich diets, with minimal mosquito reads. In contrast, the frugivorous *R. leschenaultii* contained primarily fruit flies and planthoppers, reflecting a more plant‐associated foraging niche. Low‐level fungal and protozoan DNA, detected across several species, could represent incidental ingestion or environmental contamination rather than active feeding.

**TABLE 3:** Summary of major dietary taxa detected in four Sri Lankan bat species.

Prey group	*H. speoris*	*M. phillipsi*	*R. rouxii*	*R. leschenaultii*
Mosquitoes and biting midges (*Aedes, Anopheles, Armigeres, Culex, Culicoides, Phlebotomus*)	7300	—	43,100	—
Moths and butterflies (leafrollers, hawk moths, cutworms, fruit‐piercing moths, armyworms)	4700	566,000	300,000	—
Cockroaches and ants (*Blattella, Allacta, Panesthia, Polyrhachis*)	2400	—	11,900	—
Fruit flies and hoverflies (*Drosophila, Ocyptamus*)	—	—	3400	191,500
Planthoppers and leafhoppers (*Bambusiphaga*)	—	—	—	2600
Other invertebrates (midges *Chironomus*, beetles *Sternolophus*, weevils *Myllocerus*)	5400	—	5500	—
Fungi/protozoa^a^ (*Curvularia, Trichoderma, Plasmodium*)	—	3200	74,100	3200

*Note:* The table summarizes the most abundant and ecologically relevant prey groups detected in each bat species, together with their read counts. The complete taxonomic list and full read data are provided in Supplementary Table S1.

^a^ Non‐animal DNA may reflect incidental ingestion or environmental contamination.

The alluvial diagram (Figure 1) provides a preliminary overview of prey diversity and feeding relationships among the four bat species. Distinct flows emphasize associations, such as the dominance of Bactra and Spodoptera (Lepidoptera) in *M. phillipsi* and *R. rouxii* , respectively, while narrower links indicate rare or specialized prey. Overlapping flows reveal shared dietary resources, demonstrating that some bat species exploit common prey groups despite differences in relative abundance. This integrative view highlights assumed patterns of resource partitioning and potential competition. The potential extent of dietary overlap among bat species is shown in Figure 2.

**FIGURE 1:**
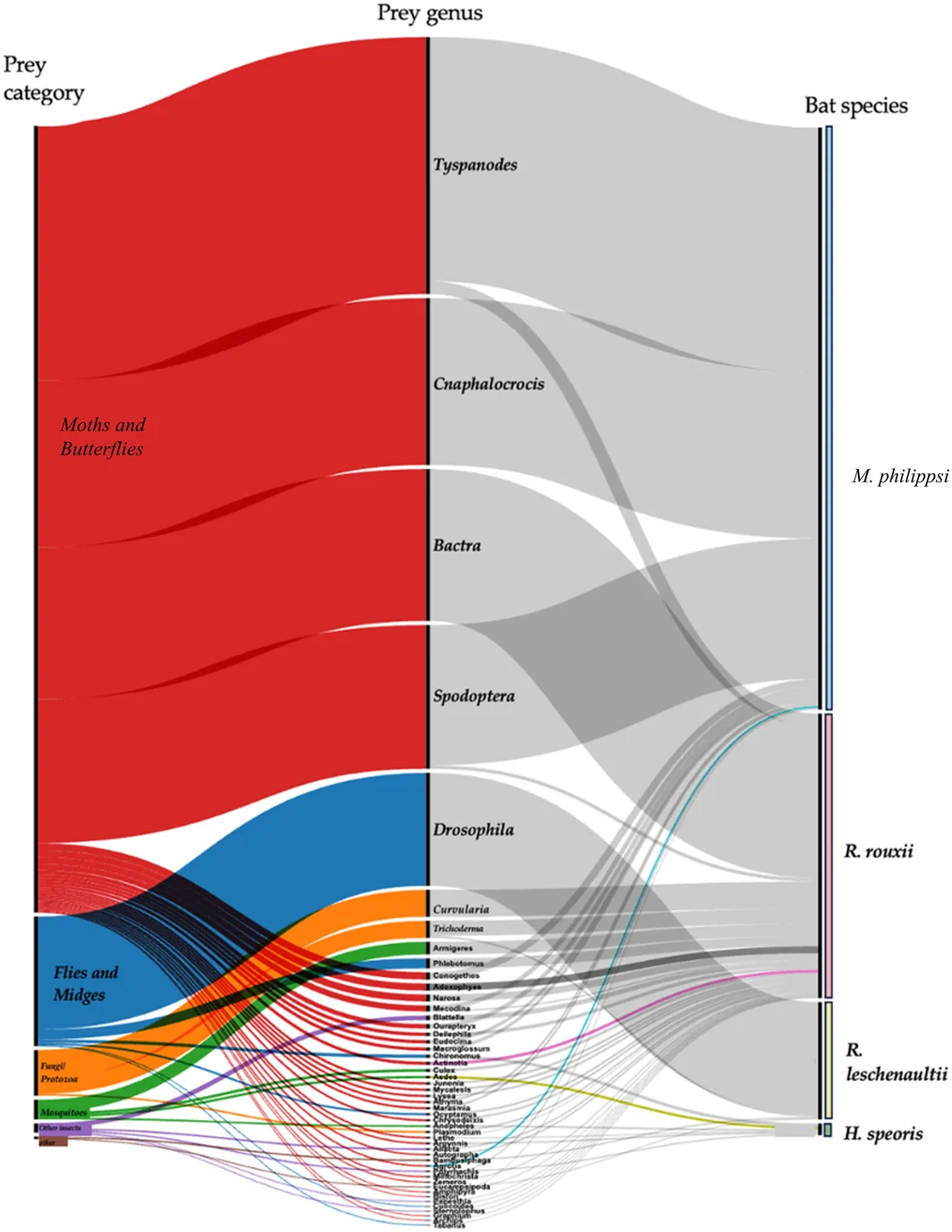
Alluvial diagram illustrating dietary composition and prey–bat associations. The diagram visualizes the flow of prey taxa across three categorical dimensions—prey Category, prey Genus, and bat Species. Each rectangle represents a unique value within a dimension, and its height is proportional to the number of sequencing reads obtained from this study. Color code: Left side columns: Moths and butterflies – red, flies and midges – blue, fungi and protozoa – orange, mosquitoes – green, other insects – purple, others – brown and right Side columns: *H. speoris* – green, *M. phillips* – blue, *R. rouxii* – red and *R. phillipsi* – yellow.

**FIGURE 2:**
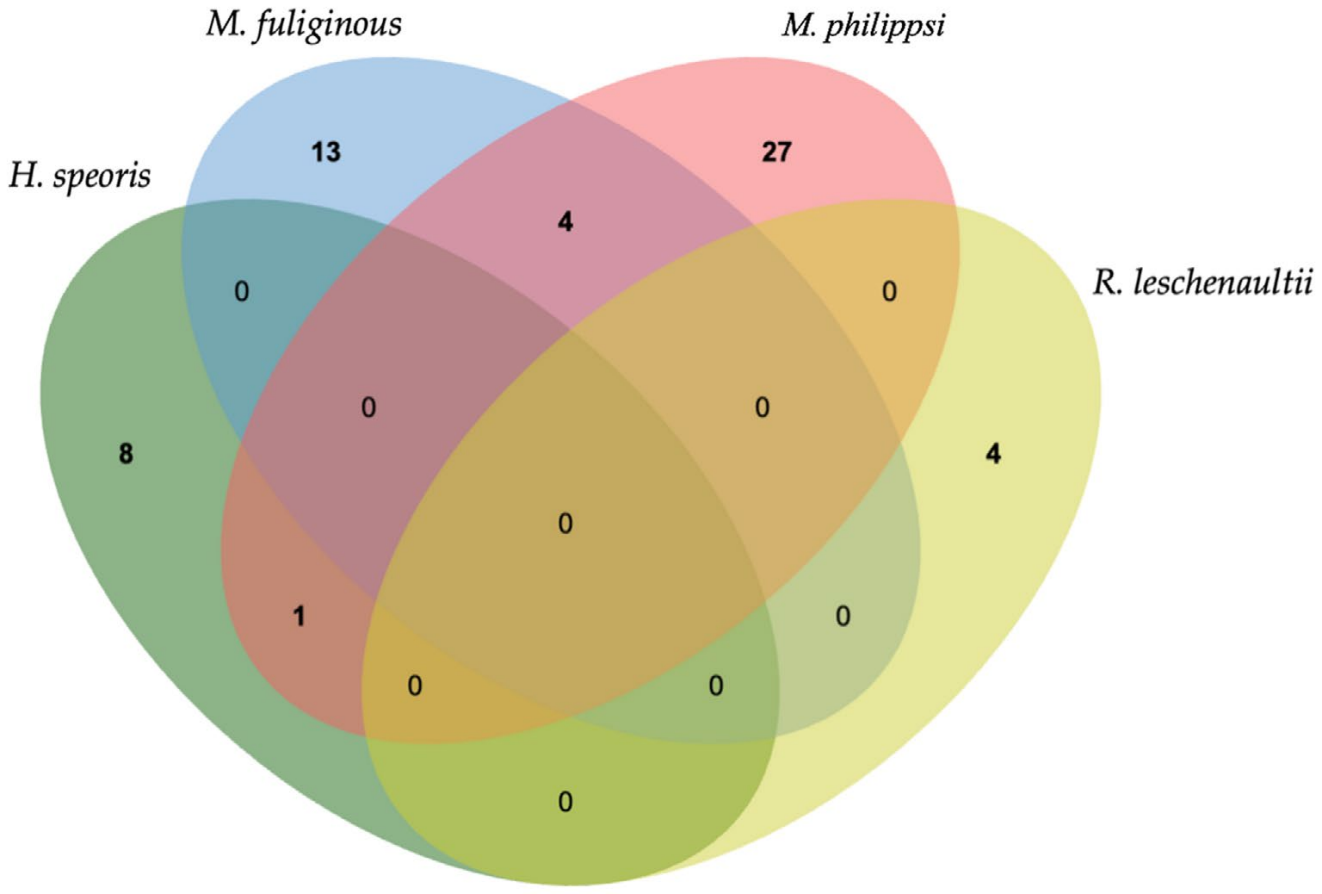
Dietary overlap among bat species occupying the Wavulgalge cave. *H. speoris, M. phillipsi, R. rouxii* and *R. leschenaultii* bat species occupying the Wavulgalge cave were included in the analysis. Color code: *H. speoris* – green, *M. philipsi* – blue, *R. rouxii* – red and *R. leschenaultii* – yellow.

In Figure 1, *H. speoris* and *R. rouxii* both share genus *Lethe*, a group of butterflies commonly known as treebrowns. Further, *M. phillipsi* and *R. rouxii* share four prey genera: *Deilephila, Mecodina, Spodoptera*, and *Tyspanodes*. *Deilephila* and *Mecodina* are nocturnal moths native to Sri Lanka, with adults feeding on nectar and larvae consuming leaves. *Spodoptera*, including the invasive Fall Armyworm ( *S. frugiperda* ), is a major agricultural pest of maize, rice, and sugarcane in Sri Lanka. *Tyspanodes* moths contribute to local moth diversity, with larvae feeding on host plants and adults active at night.

## 4 Discussion

### 4.1 Niche Partitioning and Ecological Complementarity

Our analysis of fecal DNA from four sympatric bat species in Wavulgalge Cave— *H. speoris* , *M. phillipsi* , *R. rouxii* , and *R. leschenaultii* —could suggest trophic differentiation with limited quantitative dietary overlap among species. The shared consumption of certain prey genera, particularly between *M. phillipsi* and *R. rouxii* , could indicate that sympatric bats are capable of coexisting while exploiting overlapping resources. This pattern may reflect a dynamic interplay between niche partitioning and possible ecological complementarity, mechanisms widely documented in bat communities that facilitate stable coexistence and reduce interspecific competition (Salinas‐Ramos et al. 2020; Delaval et al. 2005). Niche partitioning allows species to minimize competition through differences in prey selection, foraging microhabitats, flight altitude, or temporal activity patterns (Beilke et al. 2021; Vieira and Paise 2011). Simultaneously, although overlap was quantitatively low, functional complementarity emerges at the guild level through shared exploitation of agriculturally and medically relevant prey taxa (Ramírez‐Fráncel et al. 2022). These findings point in the direction that sympatric bats could function as complementary predators, each filling a slightly different ecological role that, together, may enhance the resilience and functionality of local ecosystems.

Under classical limiting similarity theory, stable coexistence among sympatric species requires differentiation along at least one niche axis. The observed results could suggest limited support for trophic partitioning as a possible key mechanism facilitating coexistence within this cave assemblage.

Such dietary segregation minimizes resource overlap and could promote sympatric persistence. However, this study has a pilot character and further studies are necessary to understand these mechanisms.

While the pilot nature of this study, the limited and uneven sampling across species and seasons, and the lack of environmental prey availability data preclude strong inference, the observed dietary patterns could suggest niche differentiation as an important mechanism structuring this bat species in Wavulgalge Cave and build the baseline for further analysis.

### 4.2 Dietary Overlap and Agricultural Pest Control

One aspect of our results is the potentially shared predation on *Spodoptera*, *Mecodina*, *Deilephila*, and *Tyspanodes* by *M. philipsi* and *R. rouxii* . A recent fall armyworm ( *Spodoptera frugiperda* ) outbreak caused major maize losses in Sri Lanka, with Uva Province—home to Wavulgalge Cave—among the worst affected. During the 2018/19 *maha* season, 42,436 ha were cultivated and ~73% damaged, threatening smallholder livelihoods (Perera et al. 2019). This context underscores the agricultural importance of the surrounding landscape and the potential role of insectivorous bats in suppressing crop pests. Its voracious larvae feed on foliage, while adult moths disperse widely, leading to recurrent infestations (Kumar et al. 2022). Predation on both larval and adult stages by bats highlights their role as biological control agents, potentially reducing the need for chemical pesticides and associated environmental impacts.

The detection of rice pests such as *Cnaphalocrocis* and *Marasmia* further illustrates the role of bats in suppressing insects that threaten staple crops. The genus *Deilephila*, including species like *Mecodina praecipua* and *Mecodina umbrosa*, comprises nocturnal moths whose larvae feed on leaves, making them targets of significant herbivory. Predation on these taxa suggests that bats can mitigate localized herbivore pressure, benefiting plant health and productivity.

Although *Tyspanodes* species are not classified as major pests, they contribute to the diversity of Sri Lanka's moth fauna. By consuming these insects, bats maintain insect community balance, which may indirectly influence pest dynamics through predation pressure on both pest and non‐pest species. The collective dietary contribution of bats is thus crucial, highlighting their role in maintaining both ecological and agricultural stability.

The economic significance of such predation is considerable. Globally, insectivorous bats are estimated to save agriculture billions of dollars annually by reducing crop damage (Tuneu‐Corral et al. 2023; Kolkert et al. 2021). Based on literature‐reported nightly consumption rates, a 10 g insectivorous bat may consume ~3 g of insects per night. Our study extends these findings to Sri Lanka, suggesting that even small, sympatric bat species can collectively provide substantial pest suppression across multiple crops, emphasizing their role as a sustainable, naturally occurring pest management resource.

### 4.3 Vector Suppression and Public Health

Beyond agricultural benefits, bats also consume several medically relevant insects. *H. speoris* ingested *Aedes* and *Anopheles* mosquitoes, the primary vectors of dengue and malaria, while other species consumed sandflies (*Phlebotomus*) and non‐biting midges (*Chironomus*). The detection of *Plasmodium* DNA in fecal samples could suggest potential indirect interactions with vector‐borne pathogens. These results highlight a potential public health dimension, with bats acting as regulators of vector populations.

This is particularly relevant in Sri Lanka, where vector‐borne diseases continue to pose significant public health challenges (WHO, n.d.). Conserving bat populations may therefore serve the dual purpose of supporting ecosystem services and mitigating risks from vector‐borne diseases.

### 4.4 Foraging Strategies and Functional Diversity

Observed dietary differences may reflect known foraging strategies among the studied species. Results from this snapshot pilot study potentially indicate that insectivorous bats ( *H. speoris* , *M. philipsi*, *R. rouxii* ) primarily consumed flying insects across multiple orders, including Lepidoptera, Diptera, and Coleoptera (Zhu et al. 2024). Conversely, *R. leschenaultii* , which exhibits more opportunistic feeding behavior, may have consumed smaller insects such as fruit flies (*Drosophila*) and bat flies (*Eucampsipoda*). This has to be elucidated in further studies. The detection of insect DNA in *R. leschenaultii* suggests incidental or less likely facultative insect consumption, which is consistent with observations in *R. aegyptiacus* , where bats have been reported to deliberately forage for insects (Barclay et al. 2006). Insects could provide a protein supplement for frugivorous bats; however, the mechanisms of insect detection in these species remain to be fully understood (Speakman 2001).

Additionally, sampling was conducted during the dry‐season emergence period (January). Prey availability likely varies seasonally, and our results therefore represent a temporal snapshot rather than the annual dietary breadth of the bats. For further studies we suggest collecting environmental samples as control for seasonal and spatial availability of prey in fruits and the environment.

### 4.5 Methodological Considerations

Fecal DNA metabarcoding allowed dietary profiling, detecting both hard‐bodied and delicate prey that are difficult to identify using traditional gut content analysis or visual observations (de Barba et al. 2014). The method is particularly advantageous for identifying agriculturally and medically relevant taxa, as well as cryptic species otherwise undetected in ecological surveys.

However, potential biases must be acknowledged. DNA degradation during digestion may favor detection of prey with more resistant exoskeletons, and secondary ingestion through contaminated fruits or prey can introduce confounding signals (Alberdi et al. 2019). Additionally, when prey overlap occurs, bat species may target different prey life stages, habitats, or activity periods. However, metabarcoding does not distinguish prey life stages or quantify biomass consumed (Krüger et al. 2014). This fine‐scale partitioning helps explain how sympatric bats coexist.

Furthermore, dietary read counts from metabarcoding may not directly reflect prey biomass due to potential PCR amplification bias, primer mismatch, differential digestibility, retention time variation, prey body size effects, and variation in mtDNA copy number. And Metabarcoding results may reflect primer biases and secondary predation, and thus dietary interpretations should be made cautiously. This study is limited in sample size, seasonal and spatial variance, and read count. Nevertheless, this study has a pilot character and provides a snapshot and starting point for further research studies. Composite samples were used as this study was designed as an exploratory pilot investigation to produce an initial overview of dietary diversity across bat species in this sympatric colony. Pooling samples allowed maximization of taxonomic detection rates and was used in this pilot approach to test feasibility.

### 4.6 Conservation and Management Implications

The ecological roles of bats extend beyond predation. By modulating insect populations, they indirectly influence crop yields, disease vector prevalence, and nutrient cycling within ecosystems (Kasso and Balakrishnan 2013). However, bats face threats including habitat loss, roost disturbance, and pesticide exposure (Meyer et al. 2016). Recognizing bats as providers of measurable ecosystem services can enhance their conservation priority, particularly in agricultural landscapes where their predation can complement Integrated Pest Management (IPM) strategies. Aligning bat conservation with IPM programs allows for a synergistic approach: reducing pesticide usage, conserving biodiversity, and protecting key ecosystem services (Tuneu‐Corral et al. 2023).

## 5 Conclusion

Our study provides a first overview of the diets of four sympatric bat species in Sri Lanka, suggesting overlapping and maybe complementary prey. By preying on agricultural pests, invasive insects, and disease vectors, these bats contribute to vital ecosystem services with direct implications for human well‐being. Fecal DNA metabarcoding has proven to be a powerful tool for elucidating these interactions, revealing complex predator–prey relationships that are otherwise difficult to observe.

These findings highlight the ecological and economic importance of bats in Sri Lankan agroecosystems and emphasize the value of integrating biodiversity conservation with agricultural and public health management. Future research should aim to quantify predation rates, link dietary patterns with relative prey abundance and disease dynamics, and evaluate how landscape management strategies can enhance the benefits provided by bats.

Despite the limitations of this pilot study, the detection of fragile taxa such as mosquitoes and midges supports the general robustness of this approach. Future studies integrating relative prey abundance data, stable isotope analysis, the calculation of Frequency of Occurrence (FOO), Weighted Percentage of Occurrence (WPO), individual‐level sample analysis, and PERMANOVA occupancy modeling or OUT clustering, denoising, and ASV inference or acoustic foraging observations could provide more detailed estimates of predation rates and ecosystem impacts.

## Author Contributions

**Thejanee Perera:** conceptualization (equal), data curation (equal), formal analysis (equal), investigation (equal), methodology (equal), resources (equal), visualization (equal), writing – original draft (equal), writing – review and editing (equal). **Sahan Siriwardana:** conceptualization (equal), data curation (equal), investigation (equal), writing – review and editing (equal). **Inoka Perera:** data curation (equal), funding acquisition (equal), supervision (equal), writing – review and editing (equal). **Shiroma Handunnetti:** conceptualization (equal), supervision (equal), writing – review and editing (equal). **Jagathpriya Weerasena:** conceptualization (equal), supervision (equal), writing – review and editing (equal). **Gayani Premawansa:** conceptualization (equal), data curation (equal), supervision (equal), writing – review and editing (equal). **Sunil Premawansa:** conceptualization (equal), data curation (equal), supervision (equal), writing – review and editing (equal). **Wipula Yapa:** conceptualization (equal), data curation (equal), funding acquisition (equal), investigation (equal), project administration (equal), supervision (equal), writing – review and editing (equal). **Andreas Nitsche:** conceptualization (equal), data curation (equal), funding acquisition (equal), methodology (equal), project administration (equal), resources (equal), writing – review and editing (equal). **Claudia Kohl:** conceptualization (equal), data curation (equal), formal analysis (equal), funding acquisition (equal), investigation (equal), methodology (equal), project administration (equal), resources (equal), supervision (equal), validation (equal), writing – review and editing (equal).

## Funding

This research was funded by the Federal Ministry of Health, Germany (Bundesministerium für Gesundheit, BMG) under the IDEA (IDentification of Emerging Agents) project within the Global Health Protection Programme (GHPP), grant number ZMVI1‐2517‐GHP‐703 (TP01).

## Ethics Statement

All bat handling and sample collection procedures were conducted in accordance with ethical guidelines and with permits from the Department of Wildlife Conservation, Sri Lanka (Permit No. WL/3/2/05/18) and ethical clearance from the Institute of Biology, Sri Lanka (ERC IOBSL 170 01 18).

## Conflicts of Interest

The authors declare no conflicts of interest.

## Supporting information


**Supplementary Figure S1:** CB‐3 and CB‐4 primers binding to the *Cyt b* gene region of *Thelaira chrysopruinosa* bristle flies. In silico primer evaluation was conducted using Geneious Prime (v2023.2.1) application. Copyright at 2025 Thejanee Perera.

**Supplementary Table S1:** Dietary composition of four bat species based on sequencing read counts.

## Data Availability

Sequencing data generated in this study is available in the NCBI Sequence Read Archive (SRA) under accession number PRJNA1320967. *Benefit‐Sharing Statement*: Benefits from this research accrue from the sharing of our data and results on public databases as described above.
