# Supplementary material for: Dietary Differentiation Among Sympatric Sri Lankan Bats Revealed by Nanopore Metabarcoding
Source: Ecol Evol. 2026 Nov 13;16(11):e74545. doi: 10.1002/ece3.74545 (PMC13768578; doi:10.1002/ece3.74545)
Supplement: Supplementary file 1 [file ECE3-16-e74545-s001.docx]

**Supplementary Figure 1**
