# Supplementary material for: Dietary Differentiation Among Sympatric Sri Lankan Bats Revealed by Nanopore Metabarcoding
Source: Ecol Evol. 2026 Nov 13;16(11):e74545. doi: 10.1002/ece3.74545 (PMC13768578; doi:10.1002/ece3.74545)
Supplement: Supplementary file 2 [file ECE3-16-e74545-s002.docx]

**Supplementary Table 1. Dietary composition of four bat species based on sequencing read counts**

**(*Numbers represent total number of reads assigned to each taxon*)**

| **Prey Taxon** | ***H. speoris*** | ***M. phillipsi*** | ***R. rouxii*** | ***R. leschenaultii*** |
| --- | --- | --- | --- | --- |
| **Mosquitoes (Culicidae)** |  |  |  |  |
| ***Aedes*** | **4,212** | **–** | **–** | **–** |
| ***Anopheles*** | **3,044** | **–** | **–** | **–** |
| ***Armigeres*** | **–** | **–** | **21,267** | **–** |
| ***Culex*** | **–** | **–** | **4,798** | **–** |
| **Moths & Butterflies (Lepidoptera)** |  |  |  |  |
| ***Agrotis*** | **–** | **2,557** | **–** | **–** |
| ***Actinotia*** | **–** | **–** | **5,206** | **–** |
| ***Adoxophyes*** | **–** | **–** | **11,802** | **–** |
| ***Amphipyra*** | **–** | **1,562** | **–** | **–** |
| ***Archips*** | **–** | **–** | **1,125** | **–** |
| ***Argynnis*** | **2,839** | **–** | **–** | **–** |
| ***Athyma*** | **–** | **3,581** | **–** | **–** |
| ***Autographa*** | **–** | **–** | **2,680** | **–** |
| ***Bactra*** | **–** | **–** | **257,325** | **–** |
| ***Biston*** | **–** | **1,301** | **–** | **–** |
| ***Chrysodeixis*** | **–** | **–** | **3,269** | **–** |
| ***Cnaphalocrocis*** | **–** | **282,613** | **–** | **–** |
| ***Conogethes*** | **–** | **12,230** | **–** | **–** |
| ***Deilephila*** | **–** | **–** | **5,867** | **–** |
| ***Eudocima*** | **–** | **5,626** | **–** | **–** |
| ***Graphium*** | **–** | **–** | **1,194** | **–** |
| ***Junonia*** | **–** | **–** | **3,953** | **–** |
| ***Lethe*** | **1,827** | **–** | **1,134** | **–** |
| ***Lyssa*** | **–** | **–** | **3,592** | **–** |
| ***Macroglossum*** | **–** | **5,454** | **–** | **–** |
| ***Marasmia*** | **–** | **3,471** | **–** | **–** |
| ***Mecodina*** | **–** | **7,843** | **2,366** | **–** |
| ***Miltochrista*** | **–** | **2,219** | **–** | **–** |
| ***Mycalesis*** | **–** | **–** | **3,807** | **–** |
| ***Narosa*** | **–** | **–** | **11,662** | **–** |
| ***Ourapteryx*** | **–** | **7,547** | **–** | **–** |
| ***Spodoptera*** | **–** | **237,573** | **5,886** | **–** |
| ***Tyspanodes*** | **–** | **413,963** | **21,452** | **–** |
| ***Zemeros*** | **–** | **–** | **1,870** | **–** |
| **Other Insects** |  |  |  |  |
| ***Allacta*** | **–** | **–** | **2,773** | **–** |
| ***Blattella*** | **–** | **–** | **7,892** | **–** |
| ***Panesthia*** | **–** | **–** | **1,220** | **–** |
| ***Polyrhachis*** | **2,394** | **–** | **–** | **–** |
| ***Sternolophus*** | **–** | **–** | **1,205** | **–** |
| **Flies & Midges** |  |  |  |  |
| ***Chironomus*** | **5,375** | **–** | **–** | **–** |
| ***Drosophila*** | **–** | **–** | **–** | **191,455** |
| ***Ocyptamus*** | **–** | **–** | **3,400** | **–** |
| ***Culicoides*** | **–** | **–** | **1,207** | **–** |
| ***Phlebotomus*** | **–** | **–** | **16,811** | **–** |
| ***Tabanus*** | **–** | **–** | **1,064** | **–** |
| **Fungi / Protozoa†** |  |  |  |  |
| ***Curvularia*** | **–** | **–** | **45,474** | **–** |
| ***Plasmodium*** | **–** | **–** | **3,004** | **–** |
| ***Trichoderma*** | **–** | **–** | **25,654** | **3,178** |
| **Others** |  |  |  |  |
| ***Bambusiphaga*** | **–** | **–** | **–** | **2,614** |
| ***Eucampsipoda*** | **–** | **–** | **–** | **1,706** |

**† Non-animal dietary detections (fungi/protozoa) may represent incidental ingestion or environmental DNA.**
